# An optical multiple-image authentication based on computational ghost imaging and total-variation minimization

**DOI:** 10.1016/j.heliyon.2023.e17682

**Published:** 2023-06-29

**Authors:** Yaoling Zhou, Yueer Sun, Mu Yang, Junzhao Hou, Zhaolin Xiao, Asundi Anand, Liansheng Sui

**Affiliations:** a School of Computer Science and Engineering, Xi'an University of Technology, Xi'an, 710048, China; b Yonyou Network co., Ltd, Beijing, 100085, China; c Xi'an Haitang Vocational College, Xi'an, 710038, China; d d’Optron Pte Ltd, Singapore, 639798, Singapore; e Shaanxi Key Laboratory for Network Computing and Security Technology, Xi'an, 710048, China

**Keywords:** Multiple-image authentication, Computational ghost imaging, Total-variation minimization

## Abstract

An optical multiple-image authentication is suggested using computational ghost imaging and total-variation minimization. Differing from encrypting multiple images into a noise-like ciphertext directly, as described in most conventional authentication methods, the related encoded information is embedded into a cover image to avoid the attention of eavesdroppers. First, multiple images are encoded to form real-valued sequences composed of corresponding bucket values obtained by the aid of computational ghost imaging, and four sub-images are obtained by decomposing the cover image using wavelet transform. Second, measured sequences are embedded into one of the sub-images, and embedding positions are randomly selected using corresponding binary masks. To enhance the security level, a chaotic sequence is produced using logistic map and used to scramble measured intensities. Most importantly, original images with high quality can be directly recovered using total-variation minimization. The validity and robustness of the proposed approach are verified with optical experiments.

## 1 Introduction

Due to its remarkable advantage of high computing parallelism, various optical methods applied in the field of information security such as image encryption, hiding, and authentication have been proposed during the past decades [[1], [2], [3], [4]]. Among them, the double random phase encoding (DRPE) provided a new research perspective, with which a noisy ciphertext can be obtained by encrypting a plaintext image along with two statistically independent random phase-only masks. In order to enhance the ability to resist some common attacks, DRPE has been applied in various domains, including Gyrator domain [[5], [6], [7]], Fresnel domain [8,9], fractional Fourier domain [[10], [11], [12]], and so on. Furthermore, various methods based on different kinds of optical theories and technologies, such as fractional Mellin transform [13], interference [14,15], transport of intensity equation [16], hologram [17,18], data container [19], iterative algorithm [20,21], photon-counting [22], monocular depth rendering [23], holography [24], image denoising [25], single-pixel imaging [26], and binary tree-sliced pattern [27], have been reported in recent years. Because a lot of optical parameters are used as secret keys, these methods has high security.

As an intriguing technology, computational ghost imaging can provide the potential for optical information security due to its significant property, i.e., two-dimensional signals can be recorded using a bucket detector. Meanwhile, a set of real-valued measurements are considered as the ciphertext, the channel burden of transmitting secret information can be efficiently reduced. Therefore, how to apply computational ghost imaging in cryptographic systems has received more and more attention recently [[28], [29], [30], [31], [32], [33]]. With the help of structured-detection-based ghost imaging, Xiao et al. [34] suggested an optical scheme to implement authentication, in which the object wave is disturbed using diffusers and modulated with randomly generated patterns. The random mask before modulation and the modulated target are verified by nonlinear correlation. If a peak is observed, the authentication will be successful. This method is experimentally demonstrated to have the advantage of high discrimination capacity. With the help of the customized data container, Sui et al. [35] can completely reconstruct the primary image with the ciphertext, and enhance the security by considering the conditions of chaotic function as secret keys. Simulation experiments demonstrate strong robustness against the brute force attacks. According to different scenarios, Ye et al. [36] designed two approaches to generate pseudo-random patterns based on spatial multiplexing and temporal dimension for information embedding in ghost imaging, with which the trade-off between accuracy and efficiency can be achieved. However, the large data volume affects the accuracy and post-processing efficiency. To effectively prevent the ciphertext image from being intercepted, Kang et al. [37] enhanced the security level by illuminating the camouflaged image with modulated patterns, and protected the secret information by hiding them into the camouflaged image. The efficiency of image post-processing is to be verified. Based on the combination of visual cryptography and single-pixel imaging, Jiao et al. [38] proposed two schemes to extend the application of visual cryptography to more scenarios by overlaying and embedding in single-pixel imaging. Zheng et al. [39] introduced two-channel metasurface-images into the encryption and decryption processes of single-pixel imaging, where a single metasurface combined with different specific matrices can be used in different encryption processes. The use of canonical optimization increases the robustness of the reconstructed image. Differing from conventional computational ghost imaging, Zheng et al. [40] suggested an inverse computational scheme, where random patterns are computed after bucket signals are chosen. The eavesdropper can obtain the artifact image but cannot get the correct key to crack the cipher image. This scheme shows that the inverse computation scheme combined with other encryption algorithms can further enrich the encryption process to enhance security. Zhou et al. [41] encoded a watermark to obtain bucket values by using computational ghost imaging. Then, some bucket values are loaded into one of the low-frequency components. The imperceptibility of the encryption method can be greatly improved. To avoid potential security risks, Wu et al. [42] proposed a cryptographic key distribution protocol, which provides a potential research direction according to multi-party cryptographic key distribution. Since deep learning can automatically learn features from the data, it has attracted more and more researches in different scenarios. Ravi Kumar et al. [43] used deep learning networks for training and learning of face features to implement dataset classification accurately. Anil et al. [44] proposed two cascaded deep convolutional neural networks to implement multi-scale fusion to extract more recovery features from images. Mehmood et al. [45] proposed to use the Viola-Jones algorithm to crop the detection features and use lightweight deep learning to extract features for an efficient and fast retrieval matching process. To ensure the security of image retrieval, Li et al. [46] combined a deep convolutional neural network and hashing algorithm for extracting image features and used vector homomorphic encryption to improve search effectiveness. Sui et al. [47] designed a conditional adversarial network with which the noise affection according to the reconstructed image can be reduced greatly.

In the presented article, an optical authentication is suggested for multiple images based on total-variation minimization, where binary masks also are used to choose the necessary information of each image. Instead of using traditional ghost imaging, speckle patterns to illuminate the original image can be generated with a computer in computational ghost imaging, which can efficiently reduce the complexity of optical setup. Differing from embedding the sparse information, multiple original images to be authenticated are first encoded into real-valued sequences composed of measurements using computational ghost imaging in this method, and then these measurements are embedded into one of sub-images decomposed from the cover image using the discrete wavelet transform. In this process, random binary masks are generated to choose the embedding positions in the sub-image. Two kinds of algorithms are used to reconstruct original images. One is to calculate the correlation between measurements and speckle patterns, where recovered images have a lot of noise. Another is to use the optimization such as total variation minimization by augmented Lagrangian and alternating direction algorithms (TVAL3), where measured intensities and corresponding patterns are directly input into optimization process to reconstruct original images. Also, the sub-image containing measured intensities is scrambled using the chaotic sequence produced with logistic map, and random binary masks are adopted as secret keys.

The rest of this article is arranged as follows. In Section 2, the multiple-image authentication method based on the optimization algorithm TVAL3 is presented in detail. In Section 3, the effectiveness is verified by the optical experiments. Meanwhile, the robustness and security resisting against common attacks are analyzed. In Section 4, a brief summary is provided.

## 2 Related work

As an efficient approach, space multiplexing is widely used to implement the goal of authentication for multiple original images, where the individual information is integrated into the ciphertext [48]. For an original image, the decryption and authentication can be completed only when all the corresponding secret keys are correctly paired. This encryption and authentication schemes based on multiplexing have high feasibility, effectiveness and security, and improves certain defense performance, so it is suitable for the field of secure image communication. However, it should be realized that the number of original images is somewhat limited for these methods. This is because the binary masks do not overlap each other.

The vulnerability of conventional DRPE is an insurmountable problem. To deal with this problem, Deepan et al. [49] proposed to use compressed sensing modulation patterns to adjust different coding matrices, where multiple images are sampled to obtain their sparse information. Then, all the information is nonlinearly combined to form the input of DRPE. The combination of compressed sensing and spatial multiplexing in an appropriate method can provide an auxiliary space and efficiently improve the robustness of the cryptosystem. Experimental results verify that the nonlinear encryption scheme is robust enough to resist chosen-plaintext attack.

Spatial multiplexing is widely used in multiple-image encryption and authentication because it can efficiently suppress the silhouette problem, especially for optical methods using interference. Wang et al. [50] proposed an interesting method by taking samples of each original image and obtaining sparse information using its binary mask. Then, all sparse information is integrated and encrypted into two individual phase-only masks. In the authentication process, the authorized user explicitly associates binary masks with the encrypted information to reconstruct the sparse information and verifies the information of the original images. Simulated results show that this method has considerable security, because additional protection is provided for authentication.

As it is recognized by more and more researchers, using additional mechanism of information hiding in the encryption and authentication processes can further enhance the security level. Sui et al. [51] carried original images into their corresponding quick response codes, and then converted them into index images by the means of vector quantization. After these images are integrated into an interim distribution, the ciphertext image is generated using interference. The feasibility and effectiveness are analyzed with simulations. Due to high noise tolerance of quick response codes, the affection of cross-talk can be thoroughly avoided. In addition, binary masks used in these methods are treated as secret keys, which can magnify the key space greatly.

## 3 Scheme description

**Fig. 1:**
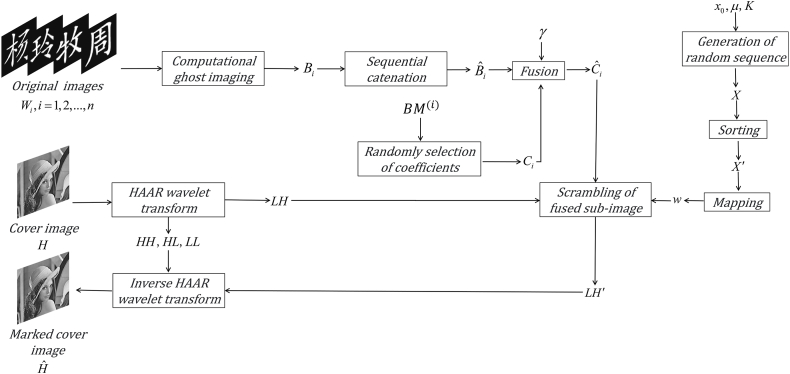
Flowchart of the embedding process of original images.

In this part, comprehensive details are given about the suggested authentication scheme based on total variation optimization. The diagram of the embedding process with the help of the discrete wavelet transform is shown in Fig. 1. Suppose that there are n images to be verified, and the i−th image is symbolized by $W_{i}\;\left(i=1,2,...,n\right)$. As illustrated in Fig. 1, the goal of achieving multi-image authentication requires three steps. First, original images are encoded into corresponding real-valued sequences. Second, the cover image is decomposed to obtain four sub-images using the discrete wavelet transform. Finally, some coefficients of one of sub-bands selected by the aid of two-dimensional binary random masks are modified by fusion between them and real-valued sequences, and the cover image carrying the information of original images is obtained with the inverse wavelet transform.

**Fig. 2:**
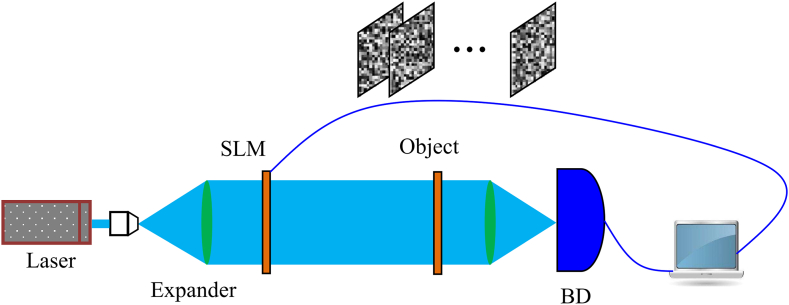
Optical setup. SLM represents the spatial light modulator, and BD represent the bucket detector.

In the first step, the optical schematic arrangement as shown in Fig. 2 is adopted to collect one-dimensional spectral data, which can be regarded as encoding original images. During data collection, a series of random or structured phase masks are loaded into spatial light modulator (SLM) sequentially. After the laser beam has undergone modulation, the corresponding structured pattern is generated and projected onto the plane where an original image $W_{j}\left(\mu ,\upsilon \right)$ is placed, namely, the original image is illuminated with this speckle pattern. Then, the intensity of transmitted wave is recorded using a bucket detector. Let $I_{i}\left(\mu ,\upsilon \right)$ represents the ith speckle pattern, the process can be mathematically denoted as

(1) $$B_{i}^{\left(j\right)}=\iint I_{i}\left(\mu ,\upsilon \right)W_{j}\left(\mu ,\upsilon \right)d\mu d\upsilon \;j=1,2,...,n$$

where $B_{i}^{\left(j\right)}\;\left(i=1,2,...,N\right)$ is the measured intensity of this original image, and N is the number of measurements. During the reconstruction, the information of $W_{j}\left(\mu ,\upsilon \right)$ is retrieved using the correlation computation between speckle patterns and its bucket values, which is mathematically described as

(2) $${\hat{W}}_{j}\left(\mu ,\upsilon \right)=\left\langle \Delta B_{i}^{\left(j\right)}\Delta I_{i}\right\rangle$$

where ⟨⋅⟩ is used to calculate the ensemble average, $\Delta B_{i}^{\left(j\right)}=B_{i}^{\left(j\right)}-\left\langle B_{i}^{\left(j\right)}\right\rangle$ and $\Delta I_{i}=I_{i}-\left\langle I_{i}\right\rangle$. Usually, in order to restore original images with high visual quality, a lots of measurements should be collected, where more and more time is consumed.

In the second step, the cover image is indicated as H, in which the measured intensities of original images will be stored, is decomposed along the horizontal and vertical directions using a discrete wavelet transform in the beginning. Four sub-images containing different frequency characteristics, i.e., the profile property and the detailed properties, are easily obtained as one low-frequency and three high-frequency components. Also, most energy is encompassed in the low-frequency component. The Haar wavelet transform has the advantages of low computational complexity and high implementation efficiency, and is widely used in the image decomposition processes in the past decades [52]. For the cover image, its four sub-images containing approximate, horizontal, vertical, and diagonal coefficients are calculated as

(3) $$\text{LL}\left(x,y\right)=\frac{1}{4}\sum_{i=0}^{1}\sum_{j=0}^{1}H\left(2x+i,2y+j\right)\text{,}$$

(4) $$\text{HL}\left(x,y\right)=\frac{1}{4}\sum_{j=0}^{1}H\left(2x,2y+j\right)-\frac{1}{4}\sum_{j=0}^{1}H\left(2x+1,2y+j\right)\text{,}$$

(5) $$\text{LH}\left(x,y\right)=\frac{1}{4}\sum_{i=0}^{1}H\left(2x+i,2y\right)-\frac{1}{4}\sum_{i=0}^{1}H\left(2x+i,2y+1\right)\text{,}$$

(6) $$\text{HH}\left(x,y\right)=\frac{1}{4}\left(H\left(2x,2y\right)+H\left(2x+1,2y+1\right)-H\left(2x+1,2y\right)-H\left(2x,2y+1\right)\right)\text{,}$$

where (x,y) is the pixel coordinate in the cover image.

In the last step, some coefficients in the suitable sub-image are chosen and fused with all measured intensities of original images to be authenticated. According to most traditional multiple-image authentication methods, the final ciphertext image is noisy. Differing from these method, all measured intensities of original images are embedded into the cover image in the proposed method. The marked cover image has high visual quality, which can avoid being noticed by saboteurs to a certain extent. Therefore, there are two competing requirements to be satisfied in the proposed method. One is the perceptual transparency of measured intensities, where the cover image cannot be destroyed with the embedding of these information. The other is to make the marked cover image robust, where some common attacks such as noise attack can be efficiently resisted. To satisfy the above requirements, some coefficients in the sub-image HL or LH are chosen based on two-dimensional binary random masks, and confused with measured intensities. Finally, the cover image $\hat{H}$ is reconstructed using the inverse wavelet transform.

It should be pointed out that, to improve the security of multiple-image authentication, the sub-image containing the information of measured intensities is confused using the chaotic sequence generated with logistic map, where the related parameters are served as secret keys with high sensitivity. Besides that, a very low number of measurements are embedded into the cover image for enhancing the security. Although no valid information is observed from its reconstruction, the corresponding authentication of this image still can be efficiently implemented. As the amount of measurement increases, the original image will be gradually reconstructed. At this moment, multiple-image encryption can be implemented. Furthermore, to solve the problem that the reconstructed result does not have high visual quality, the optimization algorithm [53], such as TVAL3, is adopted to implement the reconstruction.

As an effective approach to reduce the influence of noise in image reconstruction, TVAL3 has been widely used in the field of digital imaging. The isotropic TV model is applied to reconstruct an original image by considering its measured intensities as the observation, which is mathematically expressed as [54].

(7) $$\min_{u}TV\left(u\right)≜\sum_{i}{\left\|D_{i}u\right\|}_{2},s.t.\;Au=b$$

where $u\in R^{s}$ is the signal or image, $D_{i}u\in R^{2}$ is the discrete gradient of u at pixel i, $A\in R^{t\times s}\left(t<s\right)$ is the measurement matrix, and b is the observation of u using linear measurements. Eq. (7) is equivalent to

(8) $$\min_{w_{i}\in R^{2},u\in R^{s}}\sum_{i}{\left\|w_{i}\right\|}_{2},s.t.\;Au=b\;and\;D_{i}u=w_{i}\;for\;all\;i$$

The corresponding augmented Lagrangian problem can be described as

(9) $$\min_{w_{i},u}\sum_{i}\left({\left\|w_{i}\right\|}_{2}-\gamma_{i}^{T}\left(D_{i}u-w_{i}\right)+\frac{\beta_{i}}{2}{{\left\|D_{i}u-w_{i}\right\|}_{2}}^{2}\right)-\lambda^{T}\left(Au-b\right)+\frac{\mu }{2}{{\left\|Au-b\right\|}_{2}}^{2}$$

where γ and λ are the Lagrangian multiplier, β and μ are the penalty factor.

To solve the above problem of Eq. (8), the alternating minimization is executed, i.e., the augmented Lagrangian method and the alternating direction method are applied alternately to obtain the minimum in an iterative process. Once Eq. (9) is approximately solved within a pre-defined tolerance, the iteration will be terminated. Usually, it is preferred that the measurement matrix should have orthogonal and normalized rows. To satisfy this condition, measurements can be collected using structured patterns as described in Eq. (1), and these patterns are generated from rows of the Hadamard matrix. For an original image with S×S pixels, the Hadamard matrix with order $2^{k}$ ($S\times S=2^{k}$) can be constructed by the following recursive process. Initially, the basic matrix with order 2 is expressed as [55]

(10) $$T_{2}=\left[\begin{array}{cc} 1 & 1\\ 1 & -1 \end{array}\right]$$

Then, the matrix with order $2^{k}$, Eq. (10) is built as

(11) $$T_{2^{k}}=\left[\begin{array}{cc} T_{2^{k-1}} & T_{2^{k-1}}\\ T_{2^{k-1}} & -T_{2^{k-1}} \end{array}\right]$$

Once the matrix with the given order is obtained, a two-dimensional structured pattern with S×S pixels can be obtained by rearranging one of the row vectors.

Based on the above discussion, the embedding and the authentication process can be summarized in detail. Let H is the cover image with M×M pixels, and N measured intensities are collected for each original image $W_{i}\;\left(i=1,2,...,n\right)$. The embedding process of the multiple original image to be authenticated can be executed as follows

(1) For each original image, a total of N measured intensities are recorded using the optical setup, as depicted in Fig. 2, in which structural speckle patterns are created to illuminate the object plane. Then, a sequence containing n×N real values is obtained by the sequential catenation of measured intensities of all original images, where ${\hat{B}}_{i}\left\{i=1,2,...,n\times N\right\}$ is used to represent this sequence.
(2) Applying the Haar wavelet transform, four sub-images as described in Eqs. (3), (4), (5), (6) can be obtained from the cover image H and the size of each sub-image is M/2×M/2 pixels.
(3) Randomly generating n two-dimensional binary masks with M/2×M/2 pixels, and denoting them as $BM^{\left(i\right)}\;\left(i=1,2,...,n\right)$. In each mask, the values of N pixels in each mask are 1 and others are 0. Scanning each mask, if a pixel value equals 1, the coefficient with the same coordinate in the sub-image LH is chosen. Thus, a total of n×N coefficients can be randomly determined, and a sequence denoted as $C_{i}\left\{i=1,2,...,n\times N\right\}$ is formed by the catenation of these selected coefficients. Notably, these binary masks cannot overlap each other.
(4) Denoting γ as a real-valued attenuation factor, which is used to adjust the embedding strength of measured intensities, ${\hat{B}}_{i}\left\{i=1,2,...,n\times N\right\}$ is fused with $C_{i}\left\{i=1,2,...,n\times N\right\}$ element by element, which can be described as

(12) $${\hat{C}}_{i}=\gamma \times {\hat{B}}_{i},i=1,2,...,n\times N$$

Obviously, original coefficients of the sub-image are directly substituted with measured intensities in the proposed method. In this way, the fused sub-image is obtained by Eq. (12), which contains the information of original images to be authenticated.

(5) Giving the related parameters $x_{0}$ and μ, a sequence $X=\left\{x_{i}|i=1,2,...,\left(M/2\times M/2\right)+K\right\}$ is generated as

(13) $$x_{n+1}=p\times x_{n}\times \left(1-x_{n}\right)$$

Sorting X in the ascending order, and discarding the previous K values, the sequence $X^{\prime }=\left\{x_{w\left(i\right)}|i=1,2,...,M/2\times M/2\right\}$ is formed, and w represents the address code. Using address code, the fused sub-image generated in the previous process can be scrambled, which is denoted as $LH^{\prime }$. Notably, besides the related parameters of logistic map, the integer K also can be used as the secret key.

(6) According to LL, HL, HH, and the scrambled $LH^{\prime }$, after using the inverse Haar wavelet transform, the marked cover image $\hat{H}$ containing the information of original images is reconstructed.

**Fig. 3:**
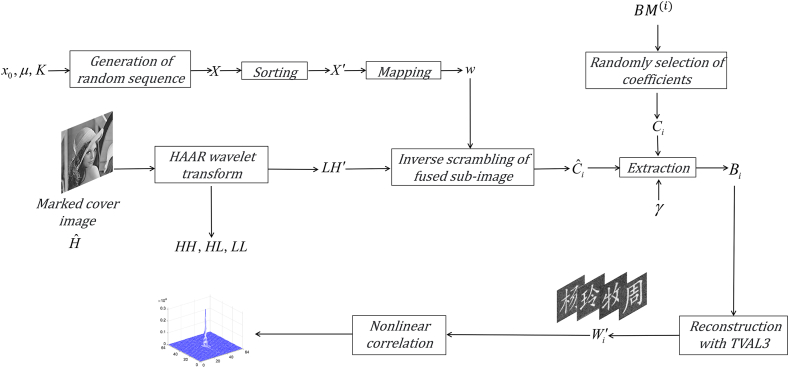
Flowchart of the authentication process of original images.

As shown in Fig. 3, the authentication process of original images can be digitally executed as follows.

(1) The marked cover image $\hat{H}$ is decomposed into LL, HL, LH, and HH using the Haar wavelet transform, where LH containing the information of original images is reserved and others are discarded.
(2) Besides the integer K, giving $x_{0}$ and μ of logistic map, the sub-image LH is inversely scrambled using the sequence generated with logistic map, which is the same as that used in the above embedding process. Thus, the original sub-image of the cover image is obtained, in which some coefficients are the modified measured intensities of original images.
(3) Scanning the secret key $BM^{\left(i\right)}$, i.e., the binary mask corresponding to the original image $W_{i}$, the pixels with modified coefficients in the sub-image LH are determined, where these values are the measured intensities adjusted with the weight factor. Therefore, the original measurements can be recovered as

(14) $$B_{i}^{\left(j\right)}=C_{i}^{\left(j\right)}/\gamma \text{,}\;i=1,2,...,N$$

where $B_{i}^{\left(j\right)}$ is the i−th recovered measurement, and $C_{i}^{\left(j\right)}$ is the corresponding modified coefficient in the sub-image.

(4) To reconstruct the original image, one intuitive way is to calculate the correlation of recovered measurements as described in Eq. (14) and speckle patterns as expressed in Eq. (2). The recovered result is noisy, especially when the number of measurements is not enough. In the proposed method, the original image $W_{i}^{\prime }$ also can be recovered based on the optimization using TVAL3, where the recovered measurements and corresponding patterns are directly used as input. Although it can relatively improve the visual quality under the condition of same number of measurements, when the number of recovered measurements is very low, the reconstructed image still is noisy, from which no valid information can be obtained.
(5) Furthermore, the nonlinear correlation map between the noisy recovered result and the original image is calculated to implement the authentication, which is mathematically expressed as [56].

(15) $$\text{NC}\left(W_{i},W_{i}^{\prime }\right)={\left|IFT\left\{{\left|FT\left\{W_{i}^{\prime }\times conj\left\{W_{i}\right\}\right\}\right|}^{p-1}FT\left\{W_{i}^{\prime }\times conj\left\{W_{i}\right\}\right\}\right\}\right|}^{2}$$

where FT{⋅} and IFT{⋅} are the Fourier transform and inverse Fourier transform, respectively, conj{⋅} is the complex conjugation, and p is the nonlinearity parameter.

In addition, when the number of recovered measurements is large, the utilization of the Gaussian filter can enhance the reconstruction performance. In the proposed method, the reconstructed image is processed with the filter with the size of 3×3 pixels.

## 4 Results and analysis

**Fig. 4:**
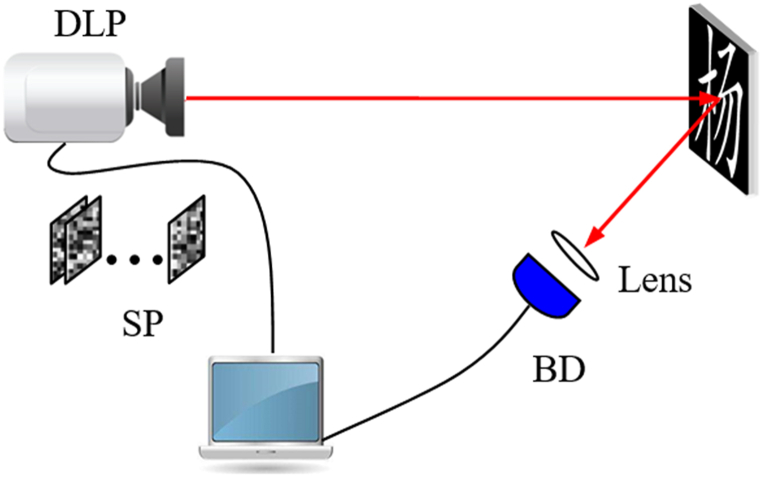
The experiment setup. DLP represents the projector, BD represents the detector to collect bucket values, and SP represents the speckle pattern.

A series of optical experiments are executed based on the optical setup as shown in Fig. 4, which is obviously different from the schematic setup. Due to the constraints of resource in our laboratory, instead of using SLM, the light modulated with structural speckle patterns and emitted from a laser projector is used to illuminate the original image. Meanwhile, the bucket detector without spatial resolution is substituted with an industrial camera to collect the total light intensity. The original image is placed on the object plane. The model of the project is XMING A62S, and the camera is IMAGING SOURVE DFK 72AUC02.

To quantitatively evaluate the performance, some important indexes such as peak signal-to-noise ratio (PSNR) and correlation coefficient (CC) are calculated. Among them, the PSNR between the cover image and its marked result is used to analyze the imperceptibility of the information of original images. If the PSNR between the cover image and the marked cover image is higher, the visual quality of the marked cover image is better. Thus, the marked cover image almost is the same as original one and the sparse information embedded will not be noticeable. Usually, it is enough that the PSNR value is larger than 30 dB. Let H and $\hat{H}$ are the cover image and the marked image, respectively. The PSNR between them can be calculated as

(16) $$\text{PSNR}\left(H,\hat{H}\right)=10\;\log_{10}\left(255^{2}M^{2}/\sum_{i=1}^{M}\sum_{j=1}^{M}{\left(H_{ij}-{\hat{H}}_{ij}\right)}^{2}\right)$$

The CC between an original image and its reconstructed result is used to evaluate the effectiveness of the proposed method, which plays a crucial role in analyzing the visual quality of the recovered image as well as the key sensitivity of the cryptosystem. Usually, the CC value can represent the degree of consistency between the original image and its reconstruction. Due to the limitation of the mechanism of imaging, the reconstructed image will be very noisy for computational ghost imaging, especially in practical experiments. In order to improve the quality, a larger number of measurements are collected. However, it will seriously reduce the efficiency of imaging. Therefore, the CC value can be used to evaluate the noisy reconstruction efficiently. Of course, the higher the CC value, the better the reconstruction. If the reconstruction is the same as original one, the maximum CC value will be 1. The CC value is mathematically calculated as

(17) $$\text{CC}\left(W_{i},W_{i}^{\prime }\right)=\frac{\sum \left(W_{i}-\bar{W}\right)\left(W_{i}^{\prime }-\bar{W^{\prime }}\right)}{\sqrt{{\sum \left(W_{i}-\bar{W}\right)}^{2}{\sum \left(W_{i}^{\prime }-\bar{W^{\prime }}\right)}^{2}}}$$

where $W_{i}$ and $W_{i}^{\prime }$ denote the pixel values in the original image and its reconstruction, respectively, $\bar{W}$ and $\bar{W^{\prime }}$ denote the corresponding average values.

**Fig. 5:**
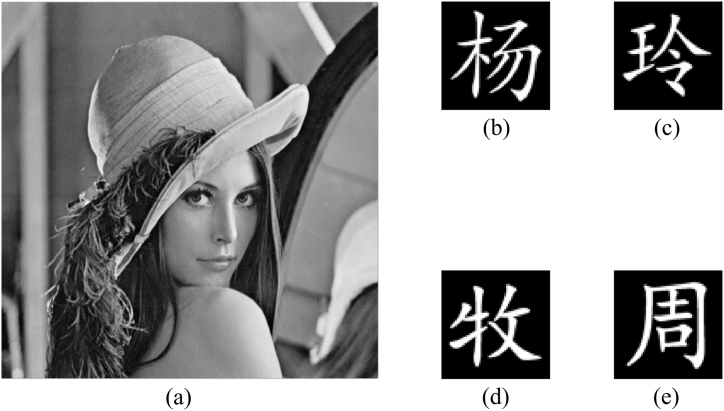
(a) The cover image “Lena”, (b)–(e) four original images to be authenticated.

**Fig. 6:**
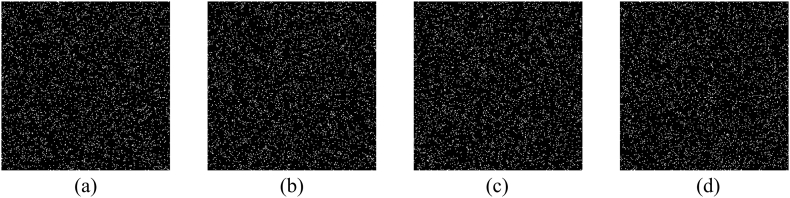
(a)–(d) Four binary masks corresponding to Fig. 5(b)–(e).

As shown in Fig. 5(a), the cover image “Lena” with 512×512 pixels is chosen from USC-SIPI image database and decomposed using the single-level Haar wavelet transform. After that, one of sub-images, i.e., the sub-image LH is utilized to embed the information of the original images. As shown in Fig. 5(b)–(e), four images with 64×64 pixels are made by ourself and will be authenticated in the following experiments. As displayed in Fig. 6(a)–(d), their corresponding binary masks with 256×256 pixels are used to randomly choose some coefficients in the sub-image. Then, these coefficients are directly substituted with the modulated measurements. Scrambling the sub-image involves generating a sequence using the logistic map in the process, the initial value $x_{0}$ is set to 0.2 and the bifurcation parameter μ is set to 3.99998. In addition, the integer parameter K is set to 2000. The weight factor γ is set to 0.000001. Because the size of the original images is 64×64 pixels, the Hadamard matrix with order $2^{12}$ is generated using Eq. (11). Thus, at most 4096 structured patterns are generated and 4096 measured intensities can be collected for each image.

**Fig. 7:**
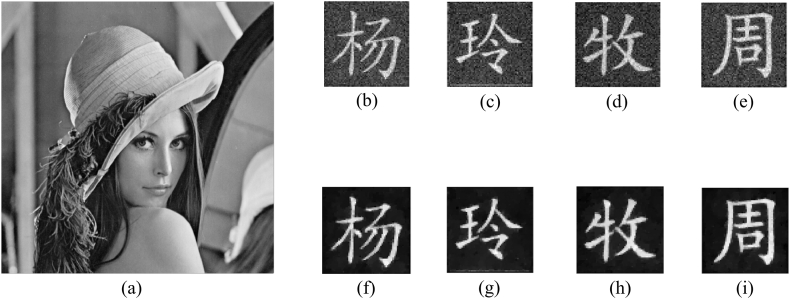
(a) The marked cover image, (b)–(e) reconstructed images using the correlation computation, and (f)–(i) reconstructed images using TVAL3.

**Fig. 8:**
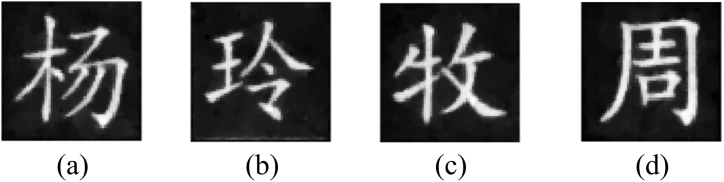
(a)–(d) Reconstruction processed using the Gaussian filter.

To verify whether the goal of multiple-image encryption can be implemented, all 4096 structured speckle patterns are generated to record measurements of all original images, and these measurements are adjusted by the weight factor and randomly embedded into the sub-image LH of the cover image based on binary masks. In Fig. 7(a), the marked cover image has high quality as the original one, and the PSNR between them is 34.3863 dB calculated using Eq. (16). The difference between them is not easily distinguished with the naked eye, which demonstrates that the imperceptibility performance of the information to be authenticated is excellent. Four images reconstructed using the correlation computation between speckle patterns and measured intensities are displayed in Fig. 7(b)–(e), where their CC values between them and corresponding original images are 0.8450, 0.8410, 0.8984 and 0.8808 by Eq. (17), respectively. At the same time, four images reconstructed using TVAL3 are displayed in Fig. 7(f)–(i), where their CC values between them and corresponding original images are 0.8966, 0.9041, 0.9423 and 0.9212, respectively. It can be seen intuitively that reconstructed results using TVAL3 have relatively high CC values, which means that original images are recovered more clearly. Furthermore, the reconstructed results can be revised with the Gaussian filter with the size of 3×3 pixels as shown in Fig. 8(a)–(d), where their corresponding CC values are 0.9010, 0.9108, 0.9422 and 0.9237, respectively.

**Fig. 9:**
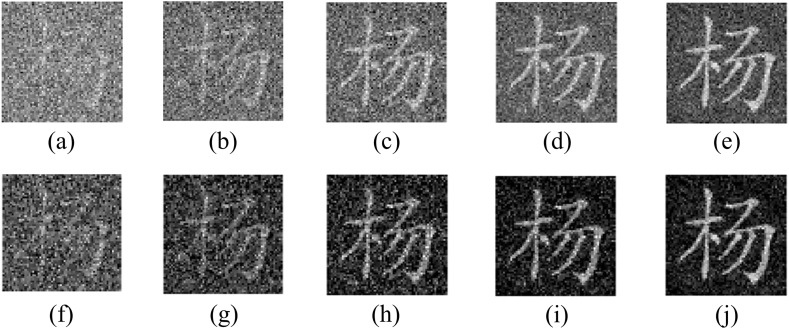
(a)–(e) First original image reconstructed using the correlation computation between speckle patterns and measured intensities, and (f)–(j) corresponding results reconstructed using TVAL3. Obviously, as the sampling ratio increases, the reconstruction becomes clearer, which can be observed in Fig. 9(a)–(e), respectively. Comparatively, the results obtained using the optimization method have high visual quality as displayed in Fig. 9(f)–(j).

**Fig. 10:**
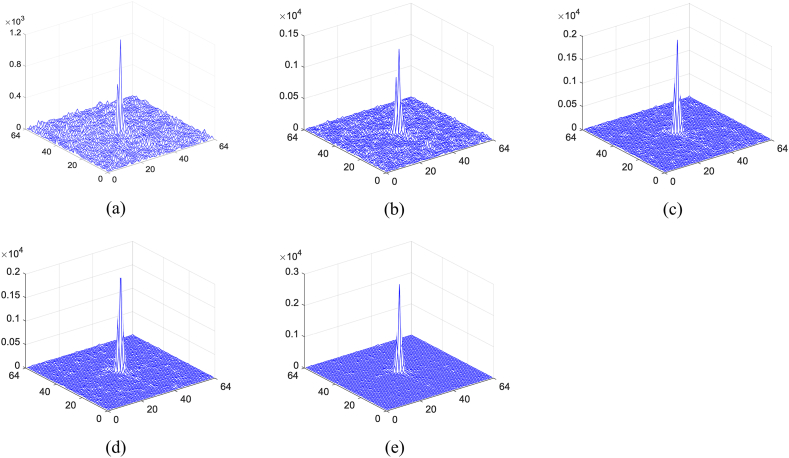
(a)–(e) Calculated nonlinear correlation map for the first original image with different sampling ratios.

According to an optical method, if only a part of measurements is collected, the efficiency of the cryptosystem can be greatly improved. However, the reconstructed result will have seriously deteriorated. Especially, when the number of measured intensities is very low, the reconstructed result will be noisy. At this moment, the original information will be verified using the correlation map calculated with Eq. (15). To analyze this function, measurements obtained at different sampling ratios are taken into account. As shown in Fig. 9(a)–(e), the first original image is reconstructed using the correlation computation between speckle patterns and measured intensities when different sampling ratios are set. Correspondingly, the results reconstructed using TVAL3 are displayed in Fig. 9(f)–(j). Obviously, with the increase of the sampling ratio, the reconstructed image becomes more and more clearly. Moreover, the results obtained using the optimization method have high visual quality. The CC and PSNR values are displayed in Table 1, where larger CC and smaller PSNR are obtained as the increase of the sampling ratio. The corresponding nonlinear correlation maps between Fig. 9(f)–(j) and the first original image are displayed in Fig. 10(a)–(e), where the nonlinearity parameter is set to 0.4. No matter what the sampling ratio is, a distinct peak can be observed in the noisy map. For other original images, similar results on reconstructed images and nonlinear correlation maps are obtained.

**Table 1:** The calculated results with different ratios.

Sampling ratio		0.1	0.2	0.4	0.6	0.8
Result using the traditional method	CC	0.2488	0.3573	0.5316	0.6526	0.7538
Result using the optimization	CC	0.3043	0.4361	0.6325	0.7398	0.8133
Marked cover image	PSNR	33.3490	32.8310	31.9731	31.2702	30.6266

As described in the previous section, three kinds of secret keys are used in order to improve the security level. First, as shown in Fig. 6, four binary masks corresponding to original images are used to embed measured intensities randomly into the sub-image of the cover image. Second, the related parameters of logistic map are used as the keys, which have high sensitivity to the tiny change. Finally, the integer parameter K is used to determine how many previous chaotic values engendered with Eq. (13) are discarded, which can ensure better randomness of the generated sequence. In the following experiments, it will be verified that no valid information will be discerned from reconstructed images if one of secret keys is not correct.

**Fig. 11:**
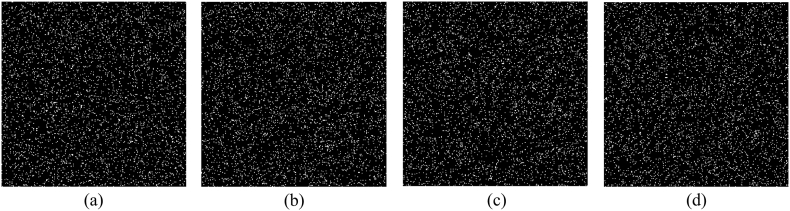
(a)–(d) Binary mask when 0.39% pixels are lost.

**Fig. 12:**
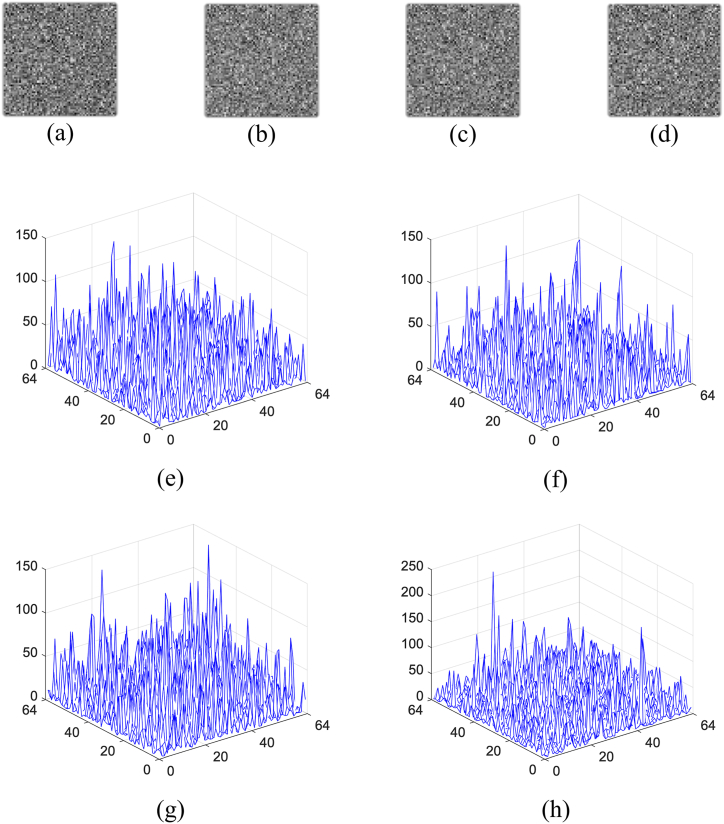
(a)–(d) Reconstruction with the binary mask in Fig. 11(a)–(d), respectively, and (e)–(h) the corresponding map.

**Fig. 13:**
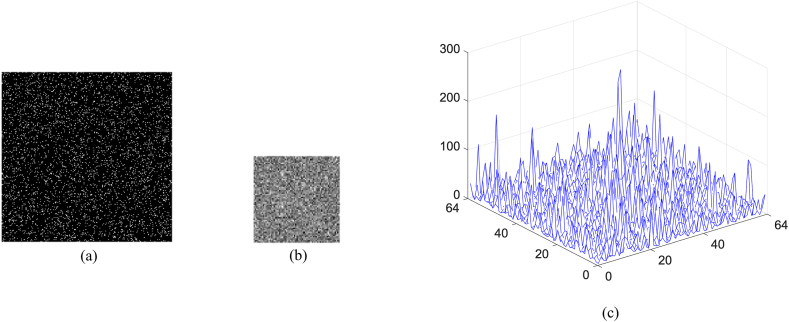
(a) 15 pixels in the third binary mask are discarded, and (b)–(c) the reconstructed image and the nonlinear correlation map.

**Fig. 14:**
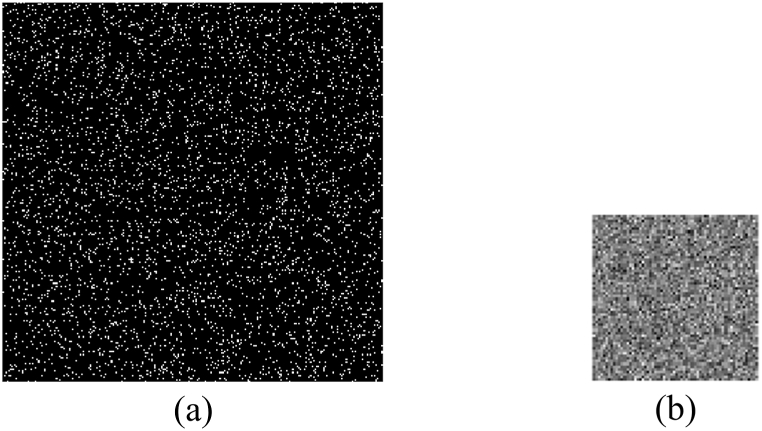
(a) The randomly generated mask and (b) the corresponding reconstruction.

**Fig. 15:**
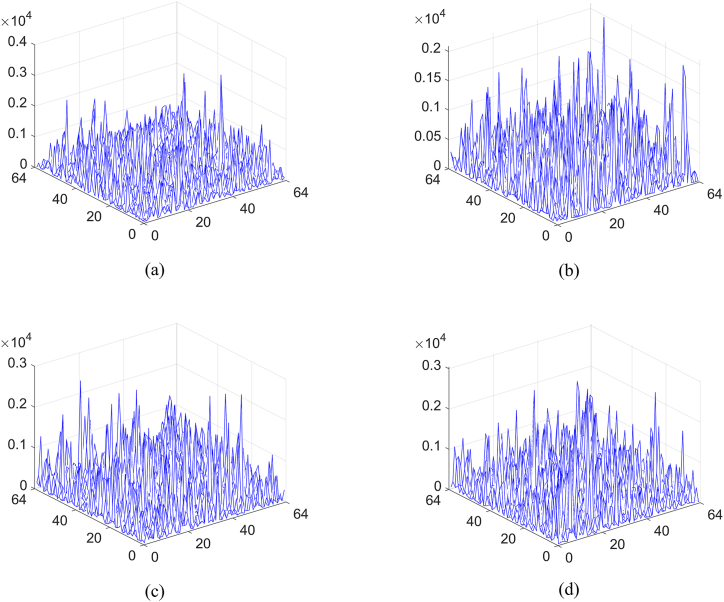
(a)–(d) The nonlinear correlation maps calculated with the reconstruction in Fig. 14(b) and four original images.

In order to verify the influence of binary masks, it is investigated that once how much data in these masks are discarded, the existence of original images cannot be authenticated. After many comparison experiments, it is found that when the number of discarded pixels of a binary mask is equal to or larger than 16, i.e., more than 0.39% of pixels, the authentication will be failure. In Fig. 11(a)-(d), 0.39% of pixels in a binary mask are discarded, that is, 16 pixels with the value of 1 are randomly lost. The obtained noisy results are displayed in Fig. 12(a)-(d), and the related maps are plotted in Fig. 12(e)–(h). Obviously, not only no information can be observed from reconstructed results, but also the authentication of original images cannot be implemented due to the lack of remarkable peaks in their nonlinear correlation maps. Conversely, if the number of discarded pixels is less than 16, an original image can be successfully authenticated. Fig. 13(a) shows the third binary mask in which 15 pixels are discarded, and the reconstructed image is displayed in Fig. 13(b). Although no valid information of the third original image can be observed in Fig. 13(b), the existence of the information can be verified as shown in Fig. 13(c), where a highest peak is located in the center. When a mask as shown in Fig. 14(a) is randomly generated and used, the image is reconstructed as shown in Fig. 14(b). The nonlinear correlation maps between it and four original images are displayed in Fig. 15(a)-(d), respectively. Obviously, the reconstructed image and all original images are uncorrelated.

**Fig. 16:**
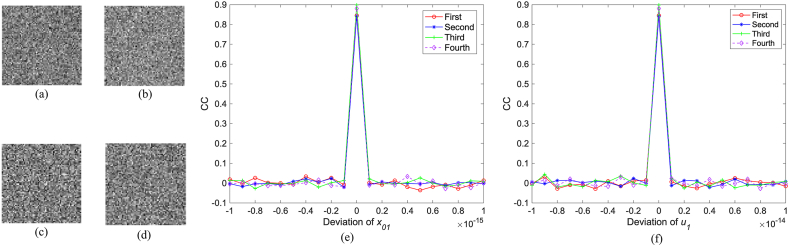
(a)–(b) Reconstruction of the first original image with $x_{0}^{\prime }=x_{0}-10^{-16}$, $x_{0}^{\prime }=x_{0}+10^{-16}$, $\mu^{\prime }=\mu -10^{-15}$, and $\mu^{\prime }=\mu +10^{-15}$, respectively, (e) the CC curve with change of the initial value, and (f) the CC curve with the change of the bifurcation parameter.

**Fig. 17:**
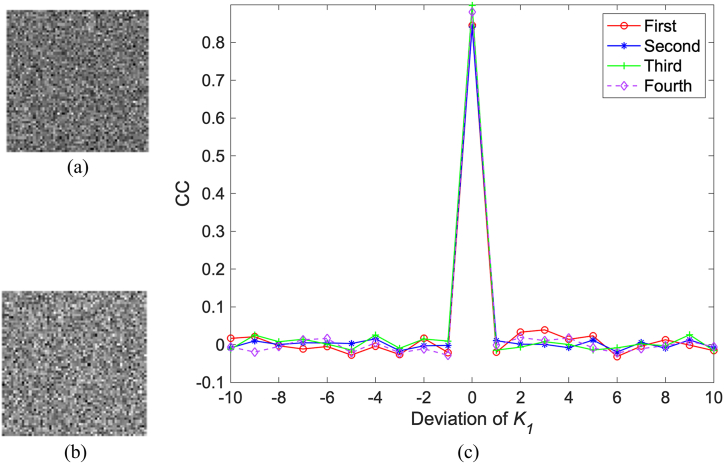
(a)–(b) Reconstruction of the first original image with $K^{\prime }=K-1$ and $K^{\prime }=K+1$, respectively, (c) the related CC curve.

Due to their high sensitivity to slight variation, the related parameters of logistic map are used as secret keys, which can potentially enhance the security. As shown in Fig. 16(a) and (b), the first original image is reconstructed only using the incorrect initial value, where the absolute deviation of $x_{0}$ is $10^{-16}$. As shown in Fig. 16(c) and (d), it is reconstructed only using the incorrect bifurcation parameter, and the absolute deviation of μ equals $10^{-15}$. It is obvious that no valid information can be observed from these results. For the initial value, the related CC curve between the first original image and its reconstruction is plotted in Fig. 16(e), from which it is known that the CC value is close to 1, otherwise it will decrease significantly for slight variation. For the bifurcation parameter, the related CC curve between them is plotted in Fig. 16(f), from which similar conclusion can be obtained. In addition, the integer parameter K as the secret key is also highly sensitive to tiny change. As shown in Fig. 17(a) and (b), the first original image is recovered when the absolute deviation of the integer parameter is 1. No valid content is observed in Fig. 17(a) and (b). The related CC curve is plotted in Fig. 17(c). It can be seen that once this integer is change, the CC value will be close to 0. Additionally, because K can be set to any integer, the key space will be very huge. For other original images, similar conclusion can be obtained. So, the proposed method has high resistance against the brute-force attack.

**Fig. 18:**
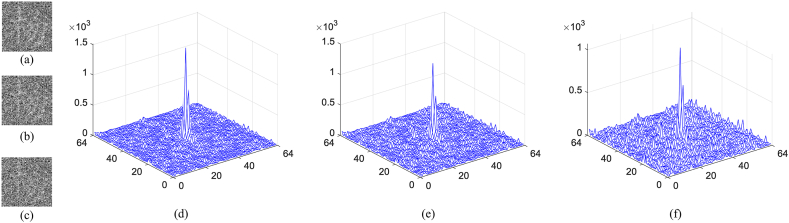
(a)–(c) First original image reconstructed with different noise strength, and (d)–(f) corresponding authentication maps.

**Fig. 19:**
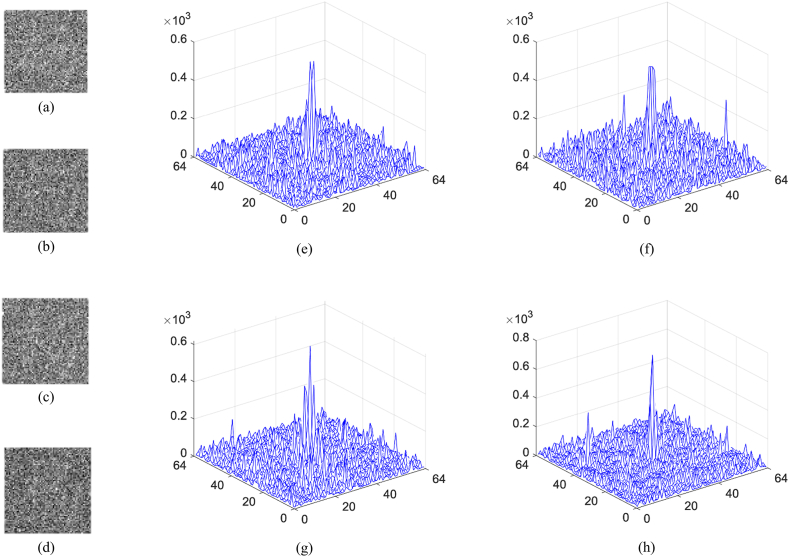
(a)–(d) First original image reconstructed in four situations, i.e., 0.78% of pixels in the marked cover image are discarded from left, right, top, and bottom, respectively, and (e)–(h) corresponding authentication maps.

To verify the robustness, the following analysis are used to investigate its resistance against noise and occlusion attacks. Let the marked cover image $\hat{H}$ is polluted with the Gaussian noise G and $\hat{H^{\prime }}$ is the polluted result, the related process can be mathematically expressed as

(18) $$\hat{H^{\prime }}=\hat{H}\times \left(1+k\times G\right)$$

where k is the noise parameter. The noise is formulated with zero-mean and 0.001 standard deviation. As shown in Fig. 18(a)–(c), the first original image is reconstructed when k is 0.03, 0.04 and 0.05 in Eq. (18), respectively. The nonlinear correlation maps for the first original image are displayed in Fig. 18(d)–(f), respectively. Although residual information are observed in these images, it is difficult to recognize the content clearly, especially when the noise strength is 0.05. However, there are remarkable peaks existed in the related maps, which efficiently demonstrate the existence of the first original image. For the occlusion attack, 0.78% of pixels in the marked cover image are discarded from left, right, top, and bottom, respectively. As shown in Fig. 19(a)–(d), the first original image is reconstructed according to four situations. It is obvious that the reconstructed results are seriously damaged. Nonetheless, the authentication of the original image still can be achieved because there are sharp peaks in the corresponding nonlinear correlation maps as shown in Fig. 19(e)–(h). For other original images, similar conclusion can be obtained. Therefore, it can be concluded that the proposed method has certain resistance according to occlusion attack.

## 5 Conclusion

An optical multiple-image authentication method using TVAL3 and wavelet transform has been proposed in this paper. After multiple images are encoded using computational ghost imaging, the resultant measurements are modulated with the weight factor and embedded into one of the sub-images decomposed from the cover image using the Haar wavelet. The marked cover image has good visual quality, and the imperceptibility of the hidden information is high, which can avoid the attention of eavesdroppers. When the sampling ratio of measurements is large, the goal of multiple-image encryption can be implemented. Otherwise, the authentication can be verified using the nonlinear correlation map. Moreover, the original image reconstructed using the optimization algorithm such as TVAL3 is better than that using correlation computation. Because multiple kinds of secret keys are applied, such as random binary masks and the related parameters of the logistic map, the security level is guaranteed. Besides that the feasibility is demonstrated, and the effectiveness of resisting malicious attacks has been verified through optical experiments.

## Funding

National Natural Science Foundation of China (NSFC): 62031023.

## Author contribution

Yaoling Zhou; Liansheng Sui: Conceived and designed the experiments; Performed the experiments; Analyzed and interpreted the data; Wrote the paper.

Yueer Sun; Mu Yang; Junzhao Hou: Analyzed and interpreted the data.

Zhaolin Xiao: Contributed reagents, materials, analysis tools or data; Wrote the paper.

Asundi Anand: Analyzed and interpreted the data; Wrote the paper.

## Data availability

Data will be made available on request.

## Declaration of competing interest

The authors declare that they have no known competing financial interests or personal relationships that could have appeared to influence the work reported in this paper.

## References

[1] Refregier P., Javidi B. (1995). Optical image encryption based on input plane and Fourier plane random encoding. Opt. Lett..

[2] Alfalou A., Brosseau C. (2015). Recent advances in optical image processing. Prog. Opt.

[3] Javidi B., Carnicer A., Yamaguchi M., Nomura T., Pérez-Cabré E., Millán M.S., Nishchal N.K., Torroba R., Barrera J.F., He W., Peng X., Stern A., Rivenson Y., Alfalou A., Brosseau C., Guo C., Sheridan J.T., Situ G., Naruse M., Matsumoto T., Juvells I., Tajahuerce E., Lancis J., Chen W., Chen X., Pinkse P.W.H., Mosk A.P., Markman A. (2016). Roadmap on optical security. J. Opt..

[4] Wang Q., Alfalou A., Brosseau C. (2017). New perspectives in face correlation research: a tutorial. Adv. Opt. Photonics.

[5] Sui L.S., Zhou B., Wang Z.M., Tian A.L. (2017). An optical color image watermarking scheme by using compressive sensing with human visual characteristics in gyrator domain. Opt Laser. Eng..

[6] Chen H., Liu Z.J., Tanougast C., Liu F.F., Blondel W. (2021). A novel chaos based optical cryptosystem for multiple images using DNA-blend and gyrator transform. Opt Laser. Eng..

[7] Abdelfattah M.G., Hegazy S.F., Areed N.F.F., Obayya S.S.A. (2022). Optical cryptosystem for visually meaningful encrypted images based on gyrator transform and Henon map. Opt. Quant. Electron..

[8] Anshula H. Singh (2021). Security-enrichment of an asymmetric optical image encryption-based devil's vortex Fresnel lens phase mask and lower upper decomposition with partial pivoting in gyrator transform domain. Opt. Quant. Electron..

[9] Yadav S., Kumar R., Singh P. (2022). Multiuser optical image authentication platform based on sparse constraint and polar decomposition in Fresnel domain. Phys. Scripta.

[10] Zhang Y.B., Zhang L., Zhong Z., Yu L., Shan M.G., Zhao Y.G. (2021). Hyperchaotic image encryption using phase-truncated fractional Fourier transform and DNA-level operation. Opt Laser. Eng..

[11] Abuturab M.R., Alfalou A. (2022). Multiple color image fusion, compression, and encryption using compressive sensing, chaotic-biometric keys, and optical fractional Fourier transform. Opt Laser. Technol..

[12] Sui L.S., Zhang X., Huang C.T., Tian A.L., Asundi A.K. (2019). Silhouette-free interference-based multiple-image encryption using cascaded fractional Fourier transforms. Opt Laser. Eng..

[13] Zhou N.R., Li H.L., Wang D., Pan S.M., Zhou Z.H. (2015). Image compression and encryption scheme based on 2D compressive sensing and fractional Mellin transform. Opt Commun..

[14] Xiong Y., Kumar R. (2022). Security analysis on asymmetric optical cryptosystem based on interference and equal modulus decomposition. Opt. Quant. Electron..

[15] Sui L.S., Du C., Zhang X., Tian A.L., Asundi A. (2019). Double-image encryption based on interference and logistic map under the framework of double random phase encoding. Opt Laser. Eng..

[16] Sui L.S., Zhao X.Y., Huang C.T., Tian A.L.A. (2019). Asundi, an optical multiple-image authentication based on transport of intensity equation. Opt Laser. Eng..

[17] Hamadi I.A., Jamal R.K., Mousa S.K. (2022). Image encryption based on computer generated hologram and Rossler chaotic system. Opt. Quant. Electron..

[18] Blau Y., Bar-on O., Hanein Y., Boag A., Scheuer J. (2020). Meta-hologram-based authentication scheme employing a speckle pattern fingerprint. Opt Express.

[19] Qin Y., Wang Z.P., Wang H.J., Gong Q., Zhou N.R. (2018). Robust information encryption diffractive-imaging-based scheme with special phase retrieval algorithm for a customized data container. Opt Laser. Eng..

[20] Moslemi V., Erfanian V., Ashoor M. (2020). Estimation of optimized timely system matrix with improved image quality in iterative reconstruction algorithm: a simulation study. Heliyon.

[21] Sui L.S., Cheng Y., Wang Z.M., Tian A.L., Asundi A.K. (2018). Single-pixel correlated imaging with high-quality reconstruction using iterative phase retrieval algorithm. Opt Laser. Eng..

[22] Lee J., Sultana N., Yi F.L., Moon I. (2018). Avalanche and bit independence properties of photon-counting double random phase encoding in gyrator domain. Curr. Opt. Photonics..

[23] Li X.W., Ren Z.Q., Guo J.F., Liu H. (2022). Light-field 3D encryption based on the monocular depth rendering. Opt. Lett..

[24] Li X.W., Wang Y., Wang Q., Kim S.T., Zhou X. (2019). Copyright protection for holographic video using spatiotemporal consistent embedding strategy. IEEE. T. Ind. Inform..

[25] Zhu Y.G., Shen W.H., Cheng F.Q., Jin C., Cao G. (2020). Removal of high density Gaussian noise in compressed sensing MRI reconstruction through modified total variation image denoising method. Heliyon.

[26] Sui L.S., Zhang L.W., Wang Q., Tian A.L., Asundi A. (2020). Multiple-image authentication based on the single-pixel correlated imaging and multiple-level wavelet transform. Opt Laser. Eng..

[27] Ravi Kumar Y.B., Narayanappa C.K., Dayananda P. (2020). Weighted full binary tree-sliced binary pattern: an RGB-D image descriptor. Heliyon.

[28] Zhang Y.D., Zhao S.M. (2017). Optical encryption scheme based on ghost imaging with disordered speckles. Chin. Phys. B.

[29] Zhu Z.J., Chi H., Tao J., Zheng S.L., Jin X.F., Zhang X.M. (2017). Single-pixel imaging based on compressive sensing with spectral-domain optical mixing. Opt Commun..

[30] Li J.B., Le M.N., Wang J., Zhang W., Li B., Peng J.Y. (2020). Object identification in computational ghost imaging based on deep learning. Appl. Phys. B.

[31] Zhang C., Zhou J.X., Tang J., Wu F., Cheng H., Sui W. (2022). Deep unfolding for singular value decomposition compressed ghost imaging. Appl. Phys. B.

[32] Sui L.S., Wang J.H., Tian A.L., Asundi A. (2019). Optical image hiding under framework of computational ghost imaging based on an expansion strategy. Opt Express.

[33] Du J., Xiong X., Quan C. (2019). High-efficiency optical image authentication scheme based on ghost imaging and block processing. Opt Commun..

[34] Xiao Y., Zhou L., Chen W. (2019). Experimental demonstration of ghost-imaging-based authentication in scattering media. Opt Express.

[35] Sui L.S., Du C., Xu M.J., Tian A.L., Asundi A. (2019). Information encryption based on the customized data container under the framework of computational ghost imaging. Opt Express.

[36] Ye Z.Y., Liu H.C., Xiong J. (2020). Computational ghost imaging with spatiotemporal encoding pseudo-random binary patterns. Opt Express.

[37] Kang Y., Zhang L.H., Ye H.L., Zhao M.T., Saima K., Zhang D.W. (2020). Camouflaged optical encryption based on compressive ghost imaging. Opt Laser. Eng..

[38] Jiao S.M., Feng J., Gao Y., Tian L., Yuan X.C. (2020). Visual cryptography in single-pixel imaging. Opt Express.

[39] Zheng P.X., Dai Q., Li Z.L., Ye Z.Y., Xiong J., Liu H.C., Zheng G.X., Zhang S. (2021). Metasurface-based key for computational imaging encryption. Sci. Adv..

[40] Zheng P.X., Tan Q.L., Liu H.C. (2021). Inverse computational ghost imaging for image encryption. Opt Express.

[41] Zhou Y.L., Yang M., Zhou B., Xiao Z.L., Sui L.S. (2022). An optical image watermarking method based on computational ghost imaging and multiple logistic maps. Appl. Phys. B Laser Opt..

[42] Yu W.K., Ning W., Li Y.X., Yang Y., Wang S.F. (2022). Multi-party interactive cryptographic key distribution protocol over a public network based on computational ghost imaging. Opt Laser. Eng..

[43] Ravi Kumar Y.B., Narayanappa C.K., Dayananda P. (2021). Assessment of facial homogeneity with regard to genealogical aspects based on deep learning approach. Turk. J. Comp. Mathem. Edu..

[44] Anil B.C., Dayananda P. (2021). Automatic liver tumor segmentation based on multi-level deep convolutional networks and fractal residual network. IETE J. Res..

[45] Mehmood I., Ullah A., Muhammad K., Deng D., Meng W., Al- Turjman F., Sajjad M., de Albuquerque V.H.C. (2019). Efficient image recognition and retrieval on IoT-assisted energy-constrained platforms from big data repositories. IEEE Internet Things J..

[46] Li S., Wu L., Meng W., Xu Z., Qin C., Wang H. (2022). DVPPIR: privacy-preserving image retrieval based on DCNN and VHE. Neural Comput. Appl..

[47] Sui L.S., Zhang L.W., Cheng Y., Xiao Z.L., Tian A.L., Asundi A. (2021). Computational ghost imaging based on the conditional adversarial network. Opt Commun..

[48] Gong Q., Liu X., Li G., Qin Y. (2013). Multiple-image encryption and authentication with sparse representation by space multiplexing. Appl. Opt..

[49] Deepan B., Quan C., Wang Y., Tay C.J. (2014). Multiple-image encryption by space multiplexing based on compressive sensing and the double-random phase encoding technique. Appl. Opt..

[50] Wang H., Qin Y., Huang Y., Wang Z., Zhang Y. (2017). Multiple-image encryption and authentication in interference-based scheme by aid of space multiplexing. Opt Laser. Technol..

[51] Sui L., Xu M., Huang C., Adhikari A., Tian A., Asundi A. (2018). Multiple-image encryption by space multiplexing based on vector quantization and interference. OSA Continuum.

[52] Sui L., Pang Z., Cheng Y., Cheng Y., Xiao Z., Tian A., Qian K.M., Asundi A. (2021). An optical image encryption based on computational ghost imaging with sparse reconstruction. Opt Laser. Eng..

[53] Gong C.M., Shao X.P., Wu T.F., Liu J.T., Zhang J.Q. (2016). Total variation optimization for imaging through turbid media with transmission matrix. Opt. Eng..

[54] Kong Q., Gong R., Liu J., Shao X. (2018). Investigation on reconstruction for frequency domain photoacoustic imaging via TVAL3 regularization algorithm. IEEE Photon. J..

[55] Wang L., Zhao S.M. (2016). Fast reconstructed and high-quality ghost imaging with fast Walsh-Hadamard transform. Photon. Res..

[56] Chen W. (2016). Correlated-photon secured imaging by iterative phase retrieval using axially-varying distances. IEEE Photon. Technol. Lett..

